# Lipids, apolipoproteins, and prognosis of amyotrophic lateral sclerosis

**DOI:** 10.1212/WNL.0000000000009322

**Published:** 2020-04-28

**Authors:** Caroline Ingre, Lin Chen, Yiqiang Zhan, Jet Termorshuizen, Li Yin, Fang Fang

**Affiliations:** From the Department of Clinical Neuroscience (C.I.), and Department of Medical Epidemiology and Biostatistics (L.C., Y.Z., J.T., L.Y., F.F.), Karolinska Institutet; and Neurology Clinic (C.I.), Karolinska University Hospital, Stockholm, Sweden.

## Abstract

**Objective:**

To determine whether lipids and apolipoproteins predict prognosis of patients with amyotrophic lateral sclerosis in a cohort study of 99 patients with amyotrophic lateral sclerosis who were diagnosed during 2015 to 2018 and followed up until October 31, 2018, at the Neurology Clinic in Karolinska University Hospital in Stockholm, Sweden.

**Methods:**

Total cholesterol, low-density lipoprotein cholesterol, high-density lipoprotein cholesterol, triglycerides, apolipoprotein AI, apolipoprotein B, and lipid ratios were measured at the time of amyotrophic lateral sclerosis diagnosis or shortly thereafter. Death after amyotrophic lateral sclerosis diagnosis was used as the main outcome. The Cox model was used to estimate hazard ratios with 95% confidence intervals of death after amyotrophic lateral sclerosis diagnosis, after controlling for sex, age at diagnosis, site of symptom onset, diagnostic delay, body mass index, Amyotrophic Lateral Sclerosis Functional Rating Scale–Revised score, and progression rate.

**Results:**

A 1-SD increase of total cholesterol (hazard ratio 0.60, 95% confidence interval 0.41–0.89, *p* = 0.01), low-density lipoprotein cholesterol (hazard ratio 0.64, 95% confidence interval 0.44–0.92, *p* = 0.02), low-density lipoprotein cholesterol/high-density lipoprotein cholesterol ratio (hazard ratio 0.65, 95% confidence interval 0.46–0.92, *p* = 0.02), apolipoprotein B (hazard ratio 0.62, 95% confidence interval 0.44–0.88, *p* = 0.01), or apolipoprotein B/apolipoprotein AI ratio (hazard ratio 0.61, 95% confidence interval 0.43–0.86, *p* < 0.01) was associated with a lower risk of death after amyotrophic lateral sclerosis diagnosis. A dose-response relationship was also noted when these biomarkers were analyzed as categorical variables.

**Conclusions:**

Lipids and apolipoproteins are important prognostic indicators for amyotrophic lateral sclerosis and should be monitored at the diagnosis of amyotrophic lateral sclerosis.

Amyotrophic lateral sclerosis (ALS) is nowadays increasingly recognized as a systemic disease affecting not only the CNS but also the whole-body physiology.^1^ Various hypotheses have been proposed concerning the potential contributions to its etiology from outside of the nervous system.^2^ Specifically, a growing body of evidence has shown dysregulated energy metabolism in ALS, which is both clinically distinct and targetable for therapeutic interventions.^3^

Increased levels of total cholesterol (TC), low-density lipoprotein cholesterol (LDL-C), triglycerides (TG), and LDL-C/high-density lipoprotein cholesterol (HDL-C) ratio have been shown to be more prevalent in patients with ALS than in controls, although results are not always consistent.^4^ In a previous study, we found that compared to controls, patients with ALS had higher levels of LDL-C, LDL-C/HDL-C, apolipoprotein (Apo) B, and ApoB/ApoAI ratio already during the 20 years before diagnosis.^4^ Using results of genome-wide association studies (GWAS) on blood lipids and ALS, in a mendelian randomization analysis, we also demonstrated evidence for a causal relationship between dyslipidemia and ALS.^5^ A causal relationship is also suggested by another 2 recent mendelian randomization analyses,^6,7^ although the inclusion of prevalent patients in the GWAS of ALS and its resultant survival bias remain a concern for these results.^8^ The potential role of lipids on ALS prognosis has also been studied, however with largely conflicting results.^9–22^ We summarize findings of the existing studies in table 1. No study has, on the other hand, examined the association of ApoB or ApoAI with ALS prognosis, however.

**Table 1:**
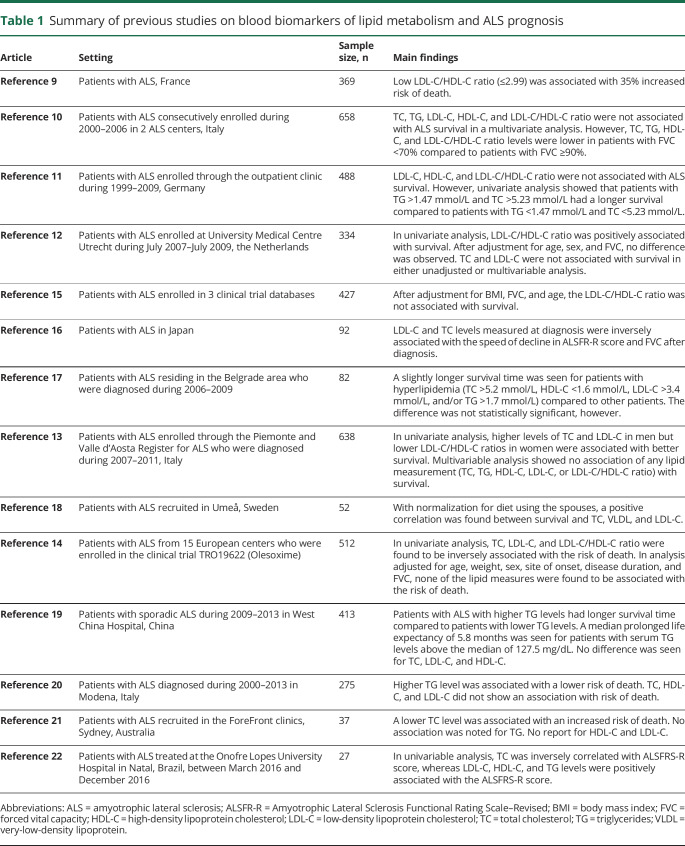
Summary of previous studies on blood biomarkers of lipid metabolism and ALS prognosis

To this end, we constructed a cohort study of 99 patients with ALS in Stockholm, Sweden, and correlated different lipids and apolipoproteins with the risk of death after ALS diagnosis, taking into account other known prognostic indicators of ALS, namely sex, age at diagnosis, site of symptom onset, diagnostic delay, body mass index (BMI), Amyotrophic Lateral Sclerosis Functional Rating Scale–Revised (ALSFRS-R) score, and progression rate.

## Methods

### Study design

We enrolled 99 patients with ALS (including 2 patients with progressive spinal muscular atrophy [PSMA]), diagnosed from September 2015 to October 2018 at the Neurology Clinic in Karolinska University Hospital in Stockholm, Sweden, for the present study. All patients were individually followed up from the date of diagnosis until death or October 31, 2018, whichever came first, through the Swedish Motor Neuron Disease Registry.^23^

We measured TC (millimoles per liter), LDL-C (millimoles per liter), HDL-C (millimoles per liter), LDL-C/HDL-C ratio, TG (millimoles per liter), ApoAI (grams per liter), ApoB (grams per liter), and ApoB/ApoAI ratio of the enrolled patients using blood samples collected after overnight fasting. The laboratory tests were conducted on fresh blood samples by the Laboratory of Karolinska University Hospital. These measurements were performed at the time of diagnosis or shortly thereafter. The mean time interval between diagnosis and lipid and apolipoprotein measurements was 1.12 months, and 87 of the patients (87.88%) had their measurements within 2 months after diagnosis.

In addition to vital status (date of death), we obtained information on sex, date of birth, age at symptom onset, site of symptom onset, age at diagnosis, BMI at diagnosis, and ALSFRS-R score at diagnosis from the Swedish Motor Neuron Disease Registry. We calculated diagnostic delay as the time interval between the time of symptom onset and date of diagnosis (in months) and progression rate as (48 − ALSFRS-R score at diagnosis)/diagnostic delay. In addition to the known prognostic indicators for ALS, we collected information on standard bicarbonate, which was measured at the same time as lipids and apolipoproteins, to assess whether the associations of lipids and apolipoproteins with ALS survival could be attributable to altered respiratory function. Overall survival time was calculated as the time interval between the date of diagnosis and date of death for deceased patients or the end of the study for the patients who were still alive at the end of 2018.

To assess the representativeness of the study sample, we compared the main clinical characteristics between the enrolled 99 patients and the remaining 118 patients who were diagnosed with ALS or PSMA during 2015 to 2018 in Stockholm according to the Swedish Motor Neuron Disease Registry but had no measurements on lipids and apolipoproteins. No clear difference was noted between these 2 groups in terms of sex, age at diagnosis, site of symptom onset, diagnostic delay, ALSFRS-R score, and progression rate (data available from Dryad, supplementary table 1, doi.org/10.5061/dryad.df02h35). Although patients included in the present study appeared to have a slightly higher BMI and mortality rate compared to patients not included, the difference was only statistically significant for BMI.

### Statistical analyses

We first described the characteristics, either as categorical or continuous variables, of the enrolled patients. Associations of lipids and apolipoproteins with the risk of death after diagnosis were assessed by hazard ratios and their 95% confidence intervals (CIs) using Cox proportional hazard regression models. In these models, we adjusted for sex (men vs women), age at diagnosis (continuous variable), site of symptom onset (bulbar vs nonbulbar), diagnostic delay (continuous variable, in months), BMI at diagnosis (continuous variable), ALSFRS-R score at diagnosis, and progression rate. Time since diagnosis was used as the underlying time scale. Because not all patients had their lipid and apolipoprotein measurements precisely at the time of diagnosis, we fitted all survival models with delayed entry at the actual time of blood sample collection, calculated as the time interval between the date of diagnosis and the date of blood sampling (in months).

We first used the studied biomarkers as continuous variables and assessed the effect of a 1-SD increase of each biomarker on the risk of death after ALS diagnosis. We then categorized these variables according to their quartiles and estimated the effect of per quartile increase of the studied biomarkers on the risk of death after ALS diagnosis. We examined the proportional hazard assumption of the Cox model by using statistics based on Schoenfeld residuals. A slight deviation of the assumption was noted for site of symptom onset and progression rate (*p* < 0.1) but not other variables. After stratification of the Cox models by these 2 covariables, the hazard ratios of other variables were nearly the same as those generated from models without such stratification. For simplicity and consistency, we chose to report findings from the original models without stratifying on these 2 variables. In the main analysis, we omitted the small number of patients with missing data in the analysis.^15^ In a first sensitivity analysis, we additionally adjusted for the analysis by standard bicarbonate, and in several other sensitivity analyses, we replaced missing data with the lowest 25th percentile, the mean, or the highest 25th percentile value of the entire cohort and compared the results of the sensitivity analyses to those of the main analysis. In another sensitivity analysis, we restricted the analysis to patients with lipids and apolipoproteins measured within 1 month after diagnosis to assess the impact of delayed blood sampling on the results. We also performed a sensitivity analysis restricting to patients with ALS alone because we included both patients with ALS and 2 patients with PSMA in the main analysis. Finally, we performed another sensitivity analysis using time since symptom onset, instead of time since diagnosis, as the underlying time scale to assess the potential influence of different time scales on the study results.

To demonstrate the relative risk of death comparing a high level to a low level of a specific biomarker, as a secondary analysis, we also dichotomized the lipids and apolipoproteins at the lowest 25th percentile for TC, HDL-C, LDL-C, ApoAI, and ApoB and at the highest 25th percentile for LDL-C/HDL-C ratio, TG, and ApoB/ApoAI ratio. These cutoffs are generally in agreement with published guidelines in cardiovascular prevention^24,25^ and apolipoprotein literature.^26^ Patients with a higher level of a specific biomarker than the cutoff were classified as having a high level of the biomarker; patients with a lower level than the cutoff were classified as having a low level of the biomarker. Kaplan-Meier survival curves were used to compare median survival time between patients of high and low levels of the studied biomarkers, and log-rank tests were used to assess the between-curve differences.

We also calculated the areas under the receiver operator characteristic curves from Cox models to select a biomarker with the greatest potential to improve the prediction of ALS prognosis.

From the findings of this analysis, we continued with a survival prediction and classified patients with ALS according to sex, age at diagnosis (below or above the mean), site of symptom onset, diagnostic delay (below or above the median), BMI (below or above the median), ALSFRS-R score (below or above the median), progression rate (below or above the median), and the selected biomarker (dichotomized as high or low).

Statistical Analysis Software (SAS Institute Inc, Cary, NC) version 9.4 was used for data analysis.

### Standard protocol approvals, registrations, and patient consents

The study was approved by the Regional Ethical Review Board in Stockholm, Sweden.

### Data availability

Researchers can apply for access to data from the present study for well-defined research questions that are in line with the overall research agenda for the cohort. Please contact the corresponding author.

## Results

Among the 99 patients, 52 were men and 47 were women (table 2). The mean age at diagnosis was 65.72 years, and the median diagnostic delay was 13 months. At the time of diagnosis, patients had a median BMI of 24.38 kg/m^2^, median ALSFRS-R score of 39, and median progression rate of 0.57. The vast majority (91.92%) of these patients used riluzole.

**Table 2:**
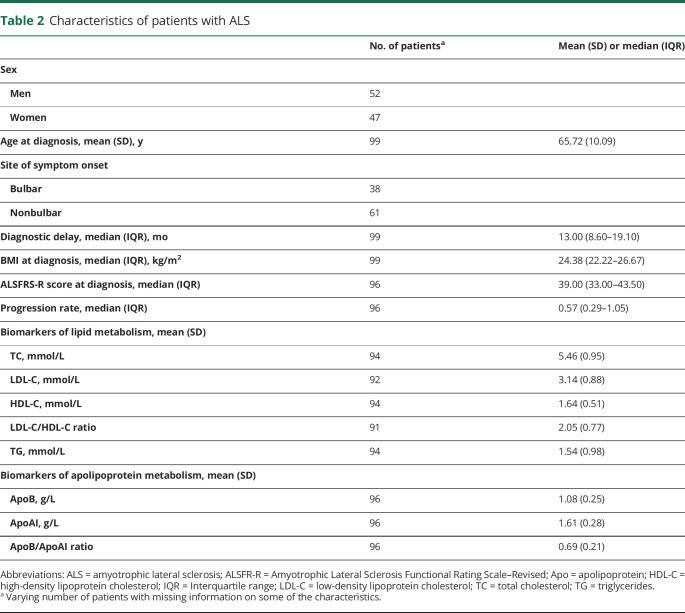
Characteristics of patients with ALS

Up to October 31, 2018, 54 patients died. The mean survival time from diagnosis to death was 13.72 months for the deceased patients. After adjustment for sex, age at diagnosis, site of symptom onset, diagnostic delay, BMI, ALSFRS-R score, and progression rate, a 1-SD increase of TC, LDL-C, LDL-C/HDL-C, ApoB, or ApoB/ApoAI ratio was statistically significantly associated with a lower risk of death after ALS diagnosis (table 3). Among the 99 patients, 95 patients had an available measurement of standard bicarbonate. Further adjustment for standard bicarbonate rendered largely similar results (data available from Dryad, supplementary table 2, doi.org/10.5061/dryad.df02h35). Sensitivity analyses using the lowest 25th percentile, mean, or highest 25th percentile values to replace missing values generated very similar results (data available from Dryad, supplementary table 3). Restricting the analysis to patients with lipid and apolipoprotein measurements within 1 month after diagnosis rendered very similar results, although with slightly limited statistical power (data available from Dryad, supplementary table 4). Restricting the analysis to patients with ALS alone similarly led to largely unchanged results (data available from Dryad, supplementary table 5). Using time since symptom onset, instead of time since diagnosis, as the underlying time scale provided results similar to those of the main analyses (data available from Dryad, supplementary table 6). Finally, using the biomarkers as categorical variables according to their quartile distributions demonstrated a similar pattern of results (table 4).

**Table 3:**
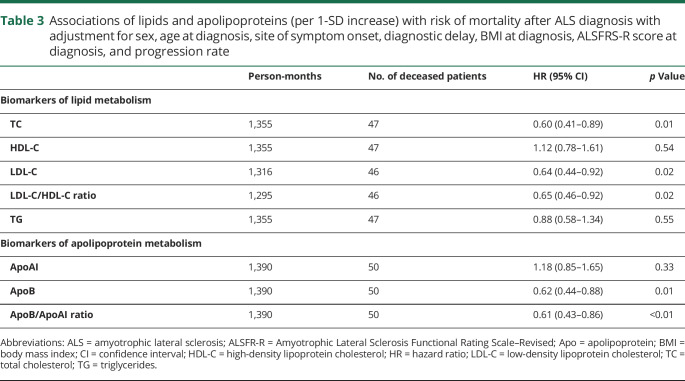
Associations of lipids and apolipoproteins (per 1-SD increase) with risk of mortality after ALS diagnosis with adjustment for sex, age at diagnosis, site of symptom onset, diagnostic delay, BMI at diagnosis, ALSFRS-R score at diagnosis, and progression rate

**Table 4:**
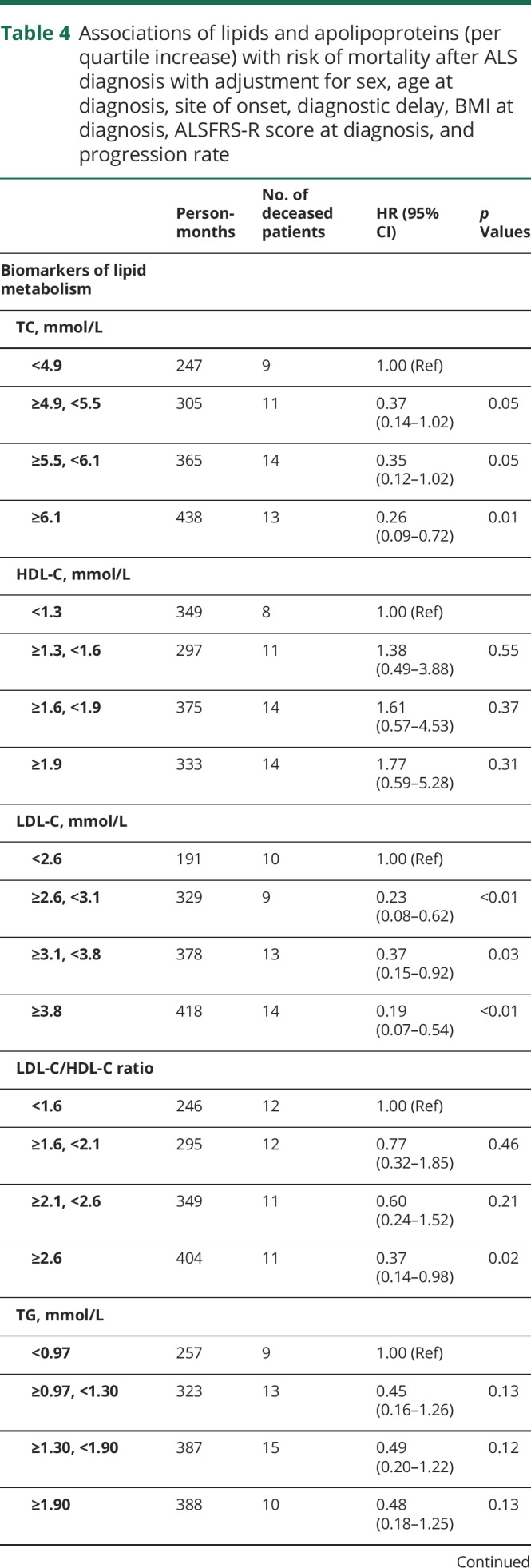

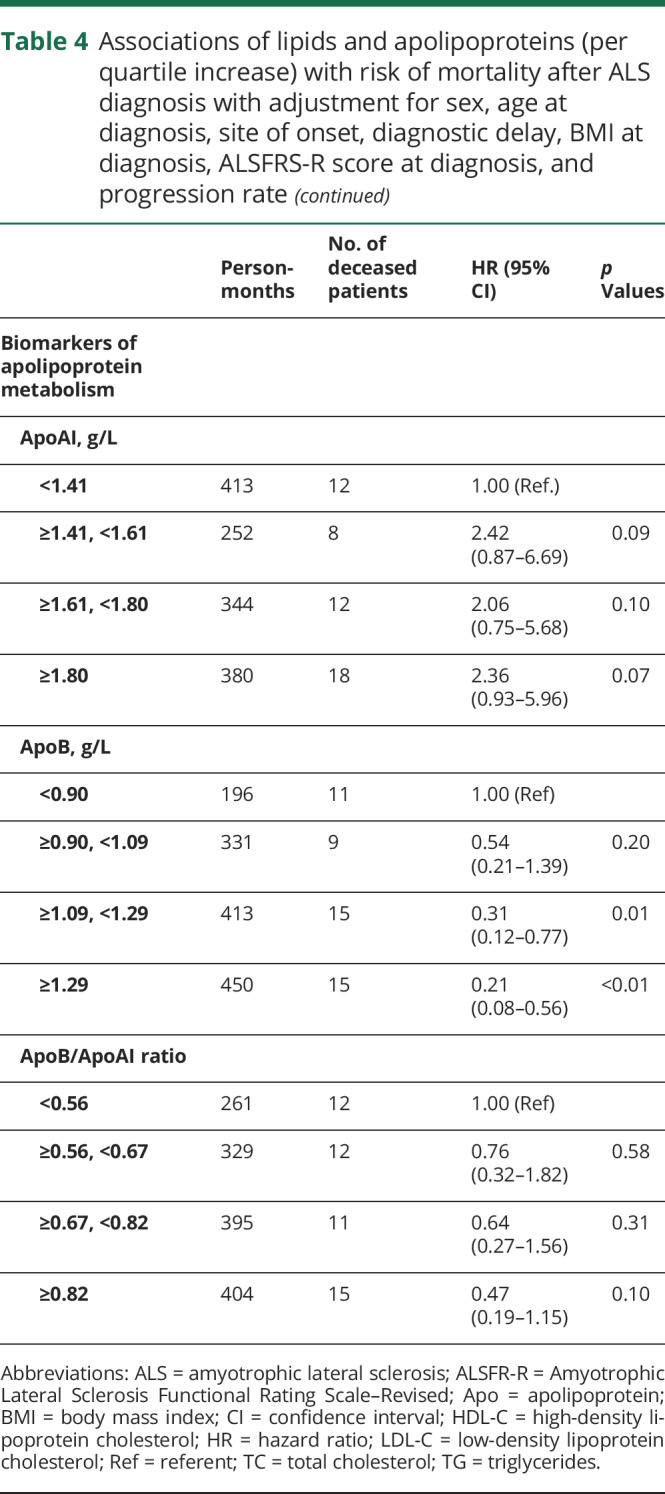
Associations of lipids and apolipoproteins (per quartile increase) with risk of mortality after ALS diagnosis with adjustment for sex, age at diagnosis, site of onset, diagnostic delay, BMI at diagnosis, ALSFRS-R score at diagnosis, and progression rate

**Figure 1:**
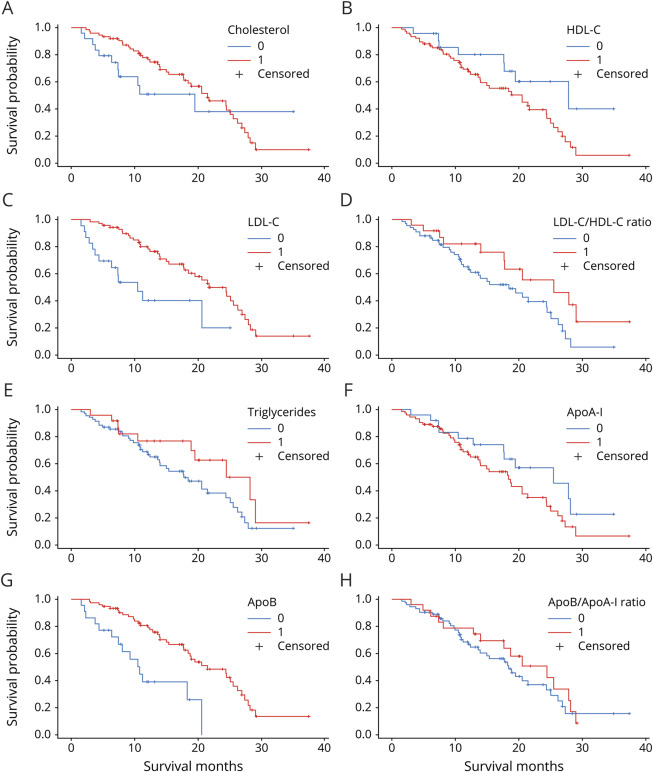
Kaplan-Meier survival curves (A) Total cholesterol ≤4.6 vs >4.6 mmol/L. Median survival 19.5 vs 21.5 months (*p* = 0.26). (B) High-density lipoprotein cholesterol (HDL-C) ≤1.4 vs >1.4 mmol/L. Median survival 27.9 vs 20.6 months (*p* = 0.06). (C) Low-density lipoprotein cholesterol (LDL-C) ≤2.6 vs >2.6 mmol/L. Median survival 10.5 vs 24.4 months (*p* < 0.01). (D) LDL-C/HDL-C ratio <2.5 vs ≥2.5. Median survival 19.5 vs 25.5 months (*p* = 0.04). (E) Triglycerides <1.8 vs ≥1.8 mmol/L. Median survival 18.4 vs 28.2 months (*p* = 0.14). (F) Apolipoprotein (Apo) AI ≤1.4 vs >1.4 g/L. Median survival 25.5 vs 18.7 months (*p* = 0.11). (G) ApoB ≤0.9 vs >0.9 g/L. Median survival 11.2 vs 21.7 months (*p* < 0.01). (H) ApoB/ApoAI ratio <0.82 vs ≥0.82. Median survival 18.8 vs 24.5 months (*p* = 0.45). Blue lines show patients with a low level of the biomarker; red lines show patients with a high level of the biomarker.

Patients with high levels of LDL-C, LDL-C/HDL-C ratio, and ApoB had a statistically significantly longer survival compared to patients with low levels of these biomarkers (figure 1). Patients with a high level of LDL-C lived 13.9 months longer than patients with a low level of LDL-C (median survival 24.4 vs 10.5 months, *p* < 0.01). Patients with a high LDL-C/HDL-C ratio lived 6 months longer compared to patients with a low LDL-C/HDL-C ratio (median survival 25.5 vs 19.5 months, *p* = 0.04). Patients with a high level of ApoB lived 10.5 months longer compared to patients with a low ApoB level (median survival 21.7 vs 11.2 months, *p* < 0.01).

**Figure 2:**
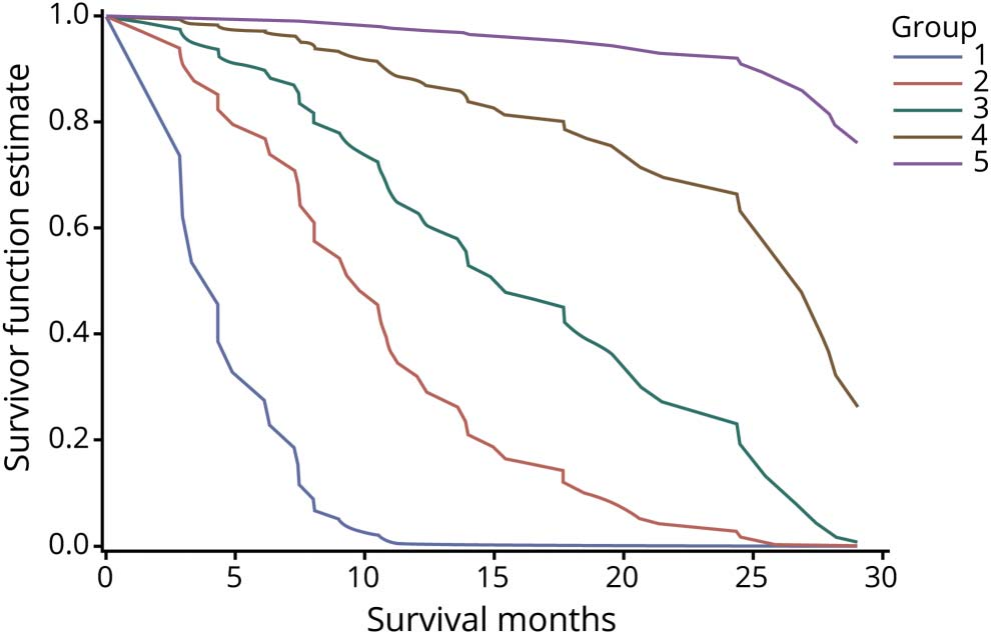
Survival prediction curves Group 1: men, bulbar onset, age at diagnosis ≥66.92 years, diagnostic delay <12.97 months, body mass index (BMI) <24.38 kg/m^2^, progression rate ≥0.57, Amyotrophic Lateral Sclerosis Functional Rating Scale–Revised (ALSFRS-R) score <39, and low-density lipoprotein cholesterol (LDL-C) ≤2.6 mmol/L. Group 2: women, bulbar onset, age at diagnosis ≥66.92 years, diagnostic delay <12.97 months, BMI <24.38 kg/m^2^, progression rate ≥0.57, ALSFRS-R score <39, and LDL-C >2.6 mmol/L. Group 3: men, bulbar onset, age at diagnosis ≥66.92 years, diagnostic delay ≥12.97 months, BMI ≥24.38 kg/m^2^, progression rate ≥0.57, ALSFRS-R score ≥39, and LDL-C >2.6 mmol/L. Group 4: men, not bulbar onset, age at diagnosis <66.92 years, diagnostic delay ≥12.97 months, BMI ≥24.38 kg/m^2^, progression rate <0.57, ALSFRS-R score ≥39, and LDL-C ≤2.6 mmol/L. Group 5: women, not bulbar onset, age at diagnosis <66.92 years, diagnostic delay ≥12.97 months, BMI ≥24.38 kg/m^2^, progression rate <0.57, ALSFRS-R score ≥39, and LDL-C >2.6 mmol/L.

Among the Cox models including any of the lipid or apolipoprotein variables, the model with LDL-C had the largest area under the curve value at 0.8853, whereas the Cox model without adding any lipid or apolipoprotein variable had an area under the curve value of 0.8303 (data available from Dryad, supplementary table 7, datadryad.org/review?doi=doi:10.5061/dryad.df02h35). We therefore decided to add LDL-C to the survival prediction model, in addition to sex, age at diagnosis, site of symptom onset, diagnostic delay, BMI, ALSFRS-R score, and progression rate. Figure 2 shows 5 exemplary survival profiles after ALS diagnosis. A male patient with a bulbar onset, age at diagnosis ≥66.92 years, diagnostic delay <12.97 months, BMI <24.38 kg/m^2^, progression rate ≥0.57, ALSFRS-R score <39, and level of LDL-C ≤2.6 mmol/L at diagnosis had the shortest predicted median survival time, whereas a female patient with a nonbulbar onset, age at diagnosis <66.92 years, diagnostic delay ≥12.97 months, BMI ≥24.38 kg/m^2^, progression rate <0.57, ALSFRS-R score ≥39, and level of LDL-C >2.6 mmol/L at diagnosis had the longest estimated survival time.

## Discussion

To the best of our knowledge, no study has previously examined both lipids and apolipoproteins as potential prognostic indicators for ALS. Using a cohort of 99 patients with ALS who were representative of all patients with ALS diagnosed during 2015 to 2018 in Stockholm, Sweden, we found that higher levels of TC, LDL-C, LDL-C/HDL-C ratio, ApoB, and ApoB/ApoAI ratio were statistically significantly associated with a lower risk of mortality, after adjustment for all other known prognostic indicators of ALS.

Several studies using diverse study populations have been conducted to examine the associations of blood lipids with the survival of patients with ALS.^9–22^ While some of the studies reported that higher lipid levels were indicative of longer survival after ALS diagnosis,^19–21^ which is in line with the present findings, other studies reported a null association between lipids and ALS survival after multivariable adjustments.^10–12,14,15^ The notable discrepancies among these studies may be attributable to the different study designs used, the varying timing of lipids measurement, and the choice of multivariable adjustments. For example, most studies recruited patients with ALS from neurology clinics for 2 distinct purposes, namely to investigate the putative risk factors for ALS using a case-control design^9,10,12,18,19,21^ or to identify and evaluate potential predictive factors for the prognosis of ALS using a prospective cohort design.^11,13–15,17,20^ In the case-control design settings, it is relatively common that not only incident cases but also prevalent cases of ALS were recruited. Because prevalent cases had to survive until study enrollment, they would have a longer survival on average compared to incident cases. Furthermore, the exposure of interest, circulating lipid levels in this case, might differ between prevalent and incident cases because lipid levels could be affected by ALS treatment or the altered lifestyle factors (e.g., diet) after ALS diagnosis. Using a prospective cohort design with incident cases of ALS only and measuring lipid levels at the time of diagnosis or immediately thereafter could largely alleviate such concerns. An additional concern for both case-control and cohort studies is the representativeness of the patients enrolled. While population-based studies are more likely to include patients of the entire spectrum of disease characteristics, clinic-based studies are more likely to include patients with specific characteristics such as younger age at onset or slower disease progression.^27^

The choice of multivariable adjustment contributed further to the conflicting results. Most of the previous studies reported a significantly improved survival of ALS diagnosis in relation to a higher level of TG, TC, LDL-C, or LDL-C/HDL-C ratio in the univariable models (e.g., nonparametric Kaplan-Meier survival curve).^9,11–15,17–21^ However, several of these studies reported that these associations diminished or disappeared after further controlling for other prognostic indicators of ALS, and the timing of the measurements of such covariables is sometimes unclear.^12,14,15^ Although it is justifiable to adjust for such covariables measured at the same time as lipids, as we did in the present study, adjustment of these variables measured later during the disease process is likely overadjustment. For example, a few studies found that LDL-C and TC levels measured at ALS diagnosis were inversely associated with the speed of decline in ALSFRS-R score and forced vital capacity after diagnosis.^16,22^ The prognostic indicators measured after lipids measurement might therefore be potential mediators that connect lipids to the survival of ALS and should not be controlled for in the multivariable-adjusted models. In our study, adjusting for standard bicarbonate concentrations measured at the same time as the studied lipids did not change the results. In addition to the above-mentioned methodologic considerations, true biological variance might also contribute to the conflicting results noted in the literature. A result pattern noted in one population, even if valid internally, does not necessarily extrapolate to an independent population of different characteristics.

In contrast to the literature on lipids and ALS prognosis, the role of apolipoproteins on ALS prognosis has rarely been described. The influence of *APOE* on the prognosis of ALS has been studied to some extent, but the findings so far did not support a strong influence of *APOE* on disease duration or overall survival.^28^ We are the first to report that high levels of ApoB and ApoB/ApoAI ratio are indicative of longer survival independently of other known prognostic indicators of ALS. Together with our recent findings of high levels of ApoB and ApoB/ApoAI ratio as potential risk factors for ALS,^4^ more research is needed to verify the present findings in independent populations and to understand the underlying mechanisms.

The strengths of the present study include the use of a study population that was highly representative of all patients with ALS diagnosed during the study period in Stockholm, the enrollment of only incident patients, the measurement of studied biomarkers at diagnosis or shortly thereafter, the rich information on clinical characteristics, and the complete follow-up for all patients included in the analysis. The comprehensive systemic review of all previously existing literature on this topic adds another layer to our study. Our study has also limitations, including the relatively small sample size, the fact that we did not include all patients diagnosed during the study period in Stockholm, and the fact that not all biomarkers were measured precisely at the time of diagnosis. We studied 99 of the total 217 patients with ALS who were eligible to participate. However, these patients did not differ clearly from the remaining 118 patients in terms of demographic and clinical characteristics (data available from Dryad, supplementary table 1, doi.org/10.5061/dryad.df02h35), suggesting a satisfactory representativeness of study sample. The similar results obtained from sensitivity analyses argue against a strong influence of slightly delayed biomarker measurements on the study results. Another limitation of the study is the lack of genetic testing for the enrolled patients, which precludes the possibility of examining the role of lipids for patients with ALS of known genetic causes.

The underlying mechanisms for the noted associations of lipids and apolipoproteins with risk of death after ALS diagnosis remain unknown. Although we had no information on forced vital capacity, the similar results obtained after further adjustment for standard bicarbonate measured at the time of diagnosis suggest that the associations are not likely confounded by respiratory function. We adjusted for BMI measured at diagnosis in all analyses, but we had no information on weight change between symptom onset and diagnosis. The contribution of weight change before diagnosis to the studied associations therefore remains to be examined. Whether the noted associations are causal is unknown. For instance, it is possible that patients with severe and more rapidly progressing ALS, because of unknown reasons, debut already lower levels of TC, LDL-C, LDL-C/HDL-C ratio, ApoB, and ApoB/ApoAI ratio at the time of diagnosis compared to other patients. The prognostic values of the studied lipids and apolipoproteins, as shown by the area under the curve values, are modest compared to other known prognostic indicators for ALS. However, for patients with specific values of other prognostic indicators, a different profile of the specific lipids and apolipoproteins at the time of diagnosis might still indicate differential prognosis. Furthermore, because of the high statistical correlations between lipids and apolipoproteins, it is difficult to disentangle the roles of apolipoproteins from the roles of lipids in the present study. Mechanistic studies are therefore needed to better understand the potential roles of apolipoproteins, especially ApoB and ApoB/ApoAI ratio, on the prognosis of ALS independently of or in interaction with lipids. For example, the brain is the most lipid-rich organ in the body, and apolipoproteins play a well-established role in the transport and metabolism of lipids within the CNS.^29^ Evidence is also emerging that apolipoproteins fulfill a number of functions beyond lipid transportation that are critical for healthy brain function.^29^ Finally, the underlying reasons for lipids (e.g., LDL-C) and apolipoproteins (e.g., ApoB) to be on the one hand potential risk factors for ALS occurrence^4–7^ but on the other hand potential protective factors for survival after ALS diagnosis, as shown in the present study, need to be studied further. If the present findings are indeed true, the protective effect on survival could have contributed partly to the noted positive association between LDL-C, ApoB, and ALS risk, assuming that patients with longer survival have higher levels of LDL-C or ApoB, are more likely to be captured and diagnosed clinically, and are more likely to be recruited in a research study for ALS (e.g., GWAS).

Lipids and apolipoproteins are important prognostic indicators for ALS and should be monitored at the diagnosis of ALS.

## Glossary

ALS: amyotrophic lateral sclerosis
ALSFR-R: Amyotrophic Lateral Sclerosis Functional Rating Scale–Revised
Apo: apolipoprotein
BMI: body mass index
GWAS: genome-wide association studies
HDL-C: high-density lipoprotein cholesterol
LDL-C: low-density lipoprotein cholesterol
PSMA: progressive spinal muscular atrophy
TC: total cholesterol
TG: triglycerides
